# Glycoform and Net Charge Heterogeneity in gp120 Immunogens Used in HIV Vaccine Trials

**DOI:** 10.1371/journal.pone.0043903

**Published:** 2012-08-22

**Authors:** Bin Yu, Javier F. Morales, Sara M. O'Rourke, Gwen P. Tatsuno, Phillip W. Berman

**Affiliations:** Department of Biomolecular Engineering, Baskin School of Engineering, University of California Santa Cruz, Santa Cruz, California, United States of America; University of Massachusetts Medical Center, United States of America

## Abstract

**Background:**

The RV144 clinical trial showed for the first time that vaccination could provide modest but significant protection from HIV-1 infection. To understand the protective response, and to improve upon the vaccine's efficacy, it is important to define the structure of the immunogens used in the prime/boost regimen. Here we examined the heterogeneity in net charge, attributable to glycoform variation, of the gp120 immunogens contained in the AIDSVAX B/E vaccine.

**Methodology/Principal Findings:**

Isoelectric focusing and glycosidase digestion were used to assess variation in net charge of the gp120s contained in the AIDSVAX B/E vaccine used in the RV144 trial. We observed 16 variants of MN-rgp120 and 24 variants of A244-rgp120. Glycoform variation in gp120 produced in Chinese hamster ovary cells was compared to glycoform variation in gp120 produced in the 293F human embryonic kidney cell line, often used for neutralization assays. We found that gp120 variants produced in CHO cells were distinctly more acidic than gp120 variants produced in 293 cells. The effect of glycoform heterogeneity on antigenicity was assessed using monoclonal antibodies. The broadly neutralizing PG9 MAb bound to A244-rgp120, but not to MN-rgp120, whether produced in CHO or in 293. However, PG9 was able to bind with high affinity to MN-rgp120 and A244-rgp120 produced in 293 cells deficient in N-acetylglucosaminyltransferase I.

**Conclusions/Significance:**

MN- and A244-rgp120 used in the RV144 trial exhibited extensive heterogeneity in net charge due to variation in sialic acid-containing glycoforms. These differences were cell line-dependent, affected the antigenicity of recombinant envelope proteins, and may affect assays used to measure neutralization. These studies, together with recent reports documenting broadly neutralizing antibodies directed against carbohydrate epitopes of gp120, suggest that glycoform variation is a key variable to be considered in the production and evaluation of subunit vaccines designed to prevent HIV infection.

## Introduction

Interest in HIV-1 envelope-based subunit vaccines has been rekindled by the results from the recent RV144 phase 3 clinical trial, involving more than 16,000 Thai volunteers [1]. In that trial, a prime-boost immunization regimen involving priming with a recombinant canarypox virus (vCP1521) encoding the gp120, gag, and protease genes from HIV-1 [2] followed by booster immunizations with the bivalent gp120 subunit vaccine, AIDSVAX B/E [3], [4], provided modest (31%) but statistically significant protection from HIV infection. This study was particularly notable since it was the first demonstration that vaccination can protect humans from HIV-1 infection. Based on these results, further studies with new vaccine antigens are planned to verify the results, optimize the immunization regimen, and collect additional information regarding correlates of protection [5]. In this regard, it is important to characterize the vaccine immunogens that elicited the protective response in the RV144 trial and to know the extent to which newly-manufactured gp120 subunit vaccines replicate their structure. The immunogens in the AIDSVAX B/E vaccine used in RV144 trial were archival preparations originally produced for the VAX003 trial [6] and methods to analyze glycogen heterogeneity have improved since the proteins were produced.

Antigenic variation has long been viewed as a major challenge in the development of a safe and effective HIV vaccine. The length of the gp120 fragment of the HIV envelope protein ranges from 484–543 amino acids in length and possesses from 18–33 N-linked predicted N-linked glycosylation sites (PNGS) [7]. Deglycosylation studies [8], [9] have shown that almost 50% of the molecular mass of the molecule can be attributed to carbohydrate. Although HIV infection is typically initiated by infection with a limited number of molecular species, the founder viruses rapidly evolve into a complex swarm of quasi-species due to relentless mutation by error-prone reverse transcriptase and selection by the immune system. The amino acid sequence of HIV can vary by 30% between individuals and as much as 10% within individuals. Besides variation in the amino acid sequence, it has long been recognized that glycosylation of the HIV-1 envelope protein represents another mechanism of antigenic variation [10], [11]. Thus, key antibody contact residues on the envelope protein are shielded from exposure to neutralizing antibodies. While the glycan shield has previously been defined by the presence or absence of carbohydrate at a particular position, the overall impact of the specific type of carbohydrate structure attached at each glycosylation site has only begun to be appreciated [12]–[16]. N-linked glycosylation in mammalian cells is complex and heterogeneous. In 1988, it was reported that N-linked carbohydrate modifications of recombinant gp120 produced in Chinese hamster ovary (CHO) cells are highly diverse and include high mannose and hybrid types of glycosylation as well as mono-, bi-, tri-, and tetra-antennary forms of complex carbohydrate leading to as many as 29 distinct glycostructures [17]. Now, it is known that as many as 64 different glycoforms can be detected at individual N-linked glycosylation sites [18]. This heterogeneity is now of particular concern due to recent findings demonstrating that the epitopes recognized by several potent broadly neutralizing (bN) monoclonal antibodies (MAbs) consist of specific carbohydrate structures on gp120 as well as specific amino acids [19]–[21].

In this paper we used two-dimensional polyacrylamide gel analysis (2D-PAGE) involving isoelectric focusing (IEF) and glycosidase digestion to visualize the heterogeneity in net charge of the two recombinant gp120 antigens, MN-rgp120 and A244-rgp120, contained in the AIDSVAX B/E vaccine. These studies provide a reference point to define the heterogeneity in net charge of the first vaccine able to provide protection from HIV-1 [1] and describe a convenient method to characterize variation in net charge and glycoform heterogeneity of the HIV envelope proteins prepared for RV144 follow-up studies.

## Materials and Methods

### Recombinant proteins

Cryopreserved archival samples of recombinant gp120 from the MN and A244 strains of HIV-1, produced in CHO cells at Genentech Inc. (S. San Francisco, CA) in the late 1990s, were obtained from Global Solutions for Infectious Diseases (GSID, S. San Francisco, CA). These proteins were incorporated into the AIDSVAX B/E vaccine [3] used in the VAX003 and RV144 Phase 3 trials [6], [1]. For purpose of comparison, A244- and MN-rgp120 were produced by transient transfection of human embryonic kidney 293F cells (Invitrogen, Carlsbad, CA). Additionally, MN-rgp120 and A244-rgp120 were produced by transient transfection of 293 cells deficient in N-acetylglucosaminyltransferase (GnTĪ) obtained from the American Type Culture Collection (ATCC, Manassas, VA). Proteins were expressed as gD fusion proteins, where a 30 amino acid flag epitope from herpes simplex virus glycoprotein D (HSV-gD) replaced the first 12 amino acids of the MN and A244 envelope proteins. Initial purification of proteins was accomplished using an immunoaffinity resin containing the same monoclonal antibody (5B6) to the N-terminus of HSV-gD used to purify the vaccine immunogens. An ion exchange chromatography polishing step using CM Sepharose (GE Healthcare Life Science, Pittsburgh, PA) was included for all protein preparations.

### Two-dimensional polyacrylamide gel electrophoresis (2D-PAGE)

Isoelectric focusing (IEF) was accomplished using the ReadyPrep^TM^ 2-D Kit (Bio-Rad Laboratories, Hercules, CA). For IEF, the recombinant proteins (150 μg) were mixed with an IEF sample buffer (185 μL) consisting of 8M urea, 2% CHAPS, 50 mM dithiothreitol (DTT), 0.2% (w/v) Bio-Lyte® 3/10 ampholytes, and trace bromophenol blue. The proteins were then loaded onto ReadyStrip^TM^ IPG strips (11 cm, pH 3–10), and resolved using a Protean® IEF Cell by a preset protocol. After IEF, the sample strips with molecular weight marker (Novex® Sharp prestained protein standard, Invitrogen) were loaded on to 4–15% polyacrylamide tris-glycine gels and run for 1 h at 200 V. SimplyBlue^TM^ SafeStain (Invitrogen) was used to stain the 2D gels. Amyloglucosidase from Aspergillus niger *(97* *kDa, pI = 3.6)* or carbonic anhydrase isozyme II (29 kDa, pI = 5.9) from bovine erythrocytes (Sigma-Aldrich Chemicals, St. Louis, MO) were used as internal standards by which the protein mobilities could be normalized. Isoelectric point standards for 2D-PAGE gels (Bio-Rad Laboratories) were used as external standards for pH gradient markers.

### Enzymatic digestion of carbohydrate

PNGase F, endoglycosidase H (Endo H), neuraminidase, and their respective reaction buffers were purchased from New England Biolabs (Ipswich, Mass). For digestion with PNGase F and Endo H, 200 μg of recombinant protein was denatured and reduced with 10X denaturation buffer and boiled at 100°C for 10 min. The samples were then mixed with 10X G7 reaction buffer and 5,000 units PNGase F, or 10X G5 reaction buffer and 5,000 units Endo H, respectively. The digestion reactions were carried out at 37°C for 24 h. For digestion with neuraminidase, 200 μg of recombinant protein was directly mixed with 10X G1 reaction buffer and 2,000 units neuraminidase, and incubated at 37°C for 24 h.

### Antibody binding assays

The human MAbs 447-52D (contributed by Susan Zolla-Pazner, New York University, New York, NY) and 17b and 48d (contributed by James Robinson, Tulane University, New Orleans, LA) were obtained through the NIH AIDS Research and Reference Reagent Program, Division of AIDS, NIAID, NIH [22], [23]. The bN MAbs 2G12 and b12 were purchased from Polymun Scientific GmbH (Vienna, Austria). The D7324 sheep polyclonal antisera to a synthetic peptide from the C5 domain was purchased from Aalto Bio Reagents (Dublin, Ireland). The PG9 MAb [24] was kindly provided by the International AIDS Vaccine Initiative, New York, NY. CD4-IgG [25] was obtained from GSID. Soluble CD4 [26] was expressed as a His-tag fusion protein in 293 cells and was purified by standard techniques. Antibody binding to recombinant gp120 was measured by an enzyme-linked immunosorbent assay (ELISA) as described previously [27]. Briefly, native rgp120 or enzyme-digested rgp120 was coated directly onto 96-well microtiter plates at 2 μg/mL in PBS. 1% BSA-PBS was used to block the plates, and serial dilutions of antibodies were added. For assays of antibodies depending on CD4-induced epitopes (48d and 17b), 10 μg/mL soluble CD4 was added to the gp120 for 1 h at 37°C before addition of serially diluted antibodies. After washing, horseradish peroxidase-labeled goat anti-human IgG or rabbit anti-goat IgG (American Qualex Antibodies, San Clemente, CA) was added to each well and incubated at 37°C for 1 h. Finally, o-phenylenediamine dihydrochloride (Sigma-Aldrich Chemicals, St. Louis, MO) solution was added and incubated at room temperature for 10 min. 3 M sulfuric acid was used to stop the reaction. The absorbance was measured at 490 nm using a SpectraMax 190 microplate reader (Molecular Devices, Sunnyvale, CA).

## Results

### Characterization of MN-rgp120 and A244-rgp120 produced in CHO and 293 cells by SDS-PAGE

**Figure 1:**
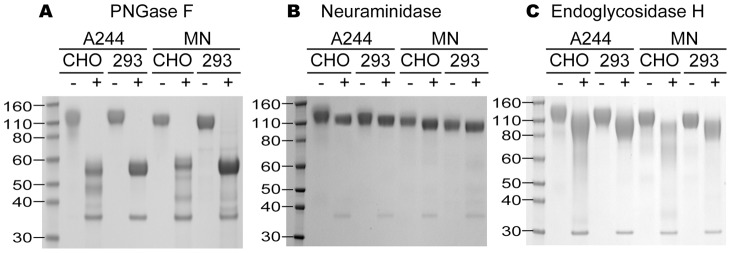
SDS PAGE analysis of MN-rgp120 and A244-rgp120 HIV produced in CHO and 293 cells. Recombinant gp120 from the MN and A244 strains of gp120 was expressed in CHO cells and in 293 cells. These were then treated with PNGase (panel A), neuraminidase (panel B), or Endo H (panel C) and resolved on 4–12% SDS-PAGE gels. Plus (+) indicates enzyme treatment; minus (−) indicates untreated.

In initial studies, one-dimensional SDS-PAGE analysis was used to compare the molecular masses of the clade B vaccine immunogen, MN-rgp120, and the CRF01_AE (clade E) vaccine immunogen, A244-rgp120, before and after glycosidase treatment to remove specific types of carbohydrates. We first compared each envelope protein as expressed in CHO and 293 cells. It can be seen in Figure 1 that untreated proteins were represented by a somewhat diffuse band in the range of 120 kDa. To assess the carbohydrate content, each of four recombinant proteins (MN CHO, MN 293, A244 CHO and A244 293) was treated with neuraminidase, Endo H, or PNGase F. N-linked glycosylation in mammalian cells is divided into two major types, a simple (high mannose) type of N-linked carbohydrate that is sensitive to degradation by Endo H, and a complex (sialic acid-containing) type of N-linked carbohydrate glycosylation that is sensitive to degradation by neuraminidase [28]. PNGase F cleaves both simple and complex N-linked carbohydrate glycosylation. The simple, high mannose form of carbohydrate begins as a tetradecasaccharide (Glc_3_Man_9_GlcNAc_2_β1, N-Asn) that is attached co-translationally, *en bloc*, to nascent proteins in the endoplasmic reticulum. This results in proteins where branched carbohydrate structures with terminal glucose and mannose residues are attached to asparagine residues within the Asn-X-Ser/Thr sequon. The simple form of glycosylation can then be converted to the complex form of glycosylation by a series of carbohydrate remodeling steps involving trimming and progressive incorporation of N-acetylglucosamine, galactose, and sialic acid by Golgi apparatus-associated glycosyltransferases. While most of the carbohydrate residues incorporated into simple and complex carbohydrate structures are uncharged, negatively charged sialic acid is often incorporated as the terminal residue of complex glycan structures. The amount of sialic acid incorporated in each N-linked glycan is known to vary from protein to protein, between cell lines, and as a function of fermentation conditions. In CHO cells, each N-linked glycosylation site typically contributes from zero to four negative charges based on incorporation of sialic acid residues [29].

We found that treatment of the four gp120s with PNGase F, that cleaves both simple and complex forms of N-linked carbohydrate, reduced the apparent mass of the four gp120s from 120 kDa to 55–60 kDa in both the MN-rgp120 and A244-rgp120 expressed in both CHO and 293 cells (Figure 1A). We also observed several discrete bands in the A244-rgp120 (e.g. 48 kDa and 38 kDa) and MN-rgp120 (e.g. 53 and 42 kDa) preparation produced in CHO cells as compared to the 293 cells. This result suggested that the archival samples had undergone some proteolytic degradation [30], with species that were only apparent following PNGase treatment. An additional lower molecular weight band (e.g. 36 kDa) on these gels was attributed to the PNGase enzyme. When these four proteins were treated with neuraminidase (Figure 1B), we observed a small but reproducible sharpening and increase in mobility of the bands associated with the enzyme-treated samples compared to the untreated samples. This result suggested that sialic acid was present in both proteins, whether expressed in 293 or CHO cells. However, because the molecular weight of sialic acid is low compared to the mass of the envelope protein, the amount of sialic acid incorporated was unclear. To assess the amount of the high mannose form present in N-linked carbohydrate, all four proteins were treated with Endo H, an enzyme that removes the simple, high mannose oligosaccharide form of N-linked carbohydrate. We found that all four proteins were also sensitive to Endo H digestion, resulting in a marked reduction of molecular weight and a broadening of the bands suggesting considerable heterogeneity in complex glycoforms (Figure 1C). These results agreed with previous studies [8], [31] showing variability in glycosylation site occupancy as well as variation in the type of glycosylation at each site and suggested that both the high mannose and complex forms of N-linked carbohydrate were present in each preparation.

### Characterization of MN-rgp120 produced in CHO and 293 cells by 2D-PAGE

**Figure 2:**
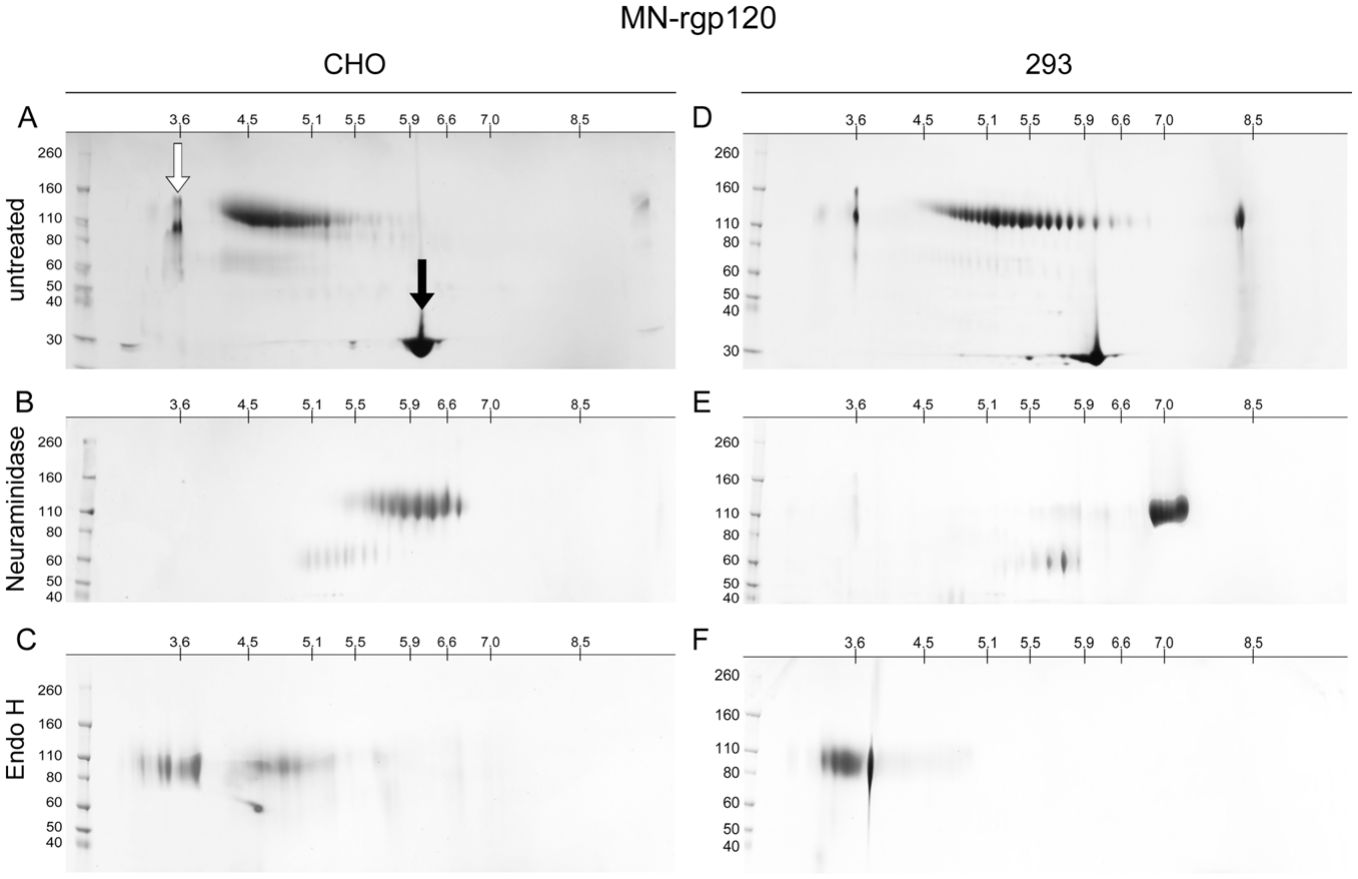
Isoelectric focusing of MN-rgp120 expressed in CHO and 293 cells with and without glycosidase digestion. The recombinant HIV-1 envelope proteins were left untreated (panels A and D), or digested with neuraminidase (panels B and E) or Endo H (panels C and F). For fractionation in the first dimension, the proteins were resolved by isoelectric focusing using 11 cm IPG strips (pH 3 to 10; Bio-Rad Laboratories, Hercules, CA). For fractionation in the second dimension, the proteins were resolved using 4–15% tris glycine SDS-PAGE gels. The specimens included amyloglucosidase (open arrow) or carbonic anhydrase isozyme II (solid arrow) as internal standards. Bands were visualized by Coomassie blue staining. Pre-stained molecular weight markers are shown to the left.

**Figure 3:**
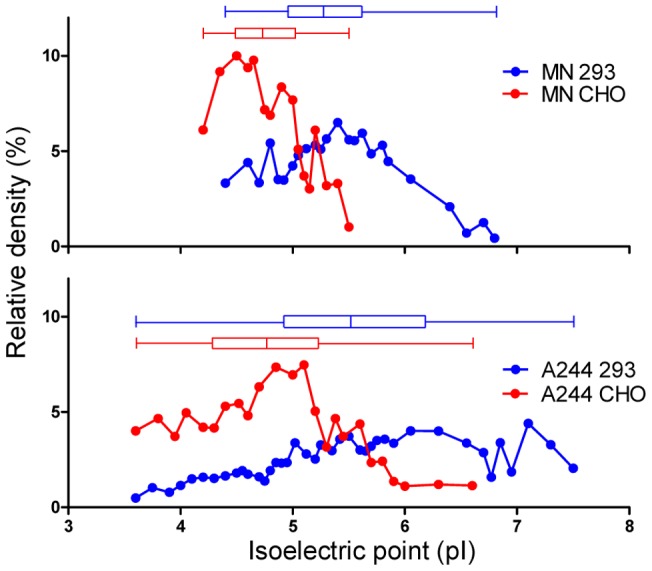
Densitometric scan of MN- and A244-rgp120s resolved by isoelectric focusing. MN-rgp120 and A244-rgp120s expressed in CHO cells or 293 cells were resolved by isolectric focusing using the Protean® isoelectric focusing system (Bio-Rad Laboratories, Hercules, CA). The gels of MN-rgp120 (Figures 2A and D) and A244-rgp120 (Figures 4A and D) were scanned using a photo-imager (FluorChem® Q, Alpha Innotech, San Leandro, CA) and plotted as a function of the observed isoelectric point (pI). Proteins produced in CHO cells are indicated by red lines; proteins produced in 293 cells are indicated by blue lines. The median, 25–75 percentile values, and the range are shown in box plots above the densitometer traces.

To explore the possibility of variation in isoelectric point (pI), we compared the MN-rgp120 proteins produced in CHO cells and 293 cells by 2D-PAGE that included IEF in the first dimension and SDS-PAGE in the second dimension. We were surprised to see that both protein preparations comprise highly heterogeneous mixtures of gp120 isoforms that varied in net charge (Figure 2A and D). This heterogeneity in net charge was far greater than what was suggested by one-dimensional SDS-PAGE analysis. Approximately 16 distinct species can be visualized in the CHO-produced MN-rgp120 preparation, whereas approximately 24 distinct molecular species can be resolved in the proteins produced in 293 cells. The pIs of both the CHO- and 293-produced MN-rgp120 proteins were far more acidic than the predicted pI (ExPASy Compute pI/MW tool) of 8.7 for non-glycosylated MN-rgp120. These results suggested that the heterogeneity in net charge observed with both proteins was due to protein modifications resulting in increased negative charge. The range of pI variation for the CHO-expressed protein was distinctly more compact and acidic than that produced in 293 cells. Densitometric scanning of the gels (Figure 3) showed that the CHO proteins possessed pIs ranging from 4.2 to 5.5 with the majority of species in the 4.2 to 5.1 range (Table 1). In contrast, the heterogeneity in pI among the proteins produced in 293 cells varied from 4.4 to 6.8, with the majority of proteins in the 4.5 to 5.9 range (Figure 3). The difference in pIs between CHO and 293 cells suggested differences in the amount of sialic acid incorporated in CHO cells compared to 293 cells (15). However, the difference in the number of species seen between the protein produced in 293 cells, as compared to protein produced in CHO cells, appeared to be due to another factor, possibly a difference in antennarity of N-linked glycoforms or a difference in the number of hybrid glycoforms [17] or variation in glycosylation site occupancy [18].

**Table 1:** The impact of glycosylation on HIV-1 gp120 isoelectric point.

Protein	Cells	Predicted pI without glycosylation	Observed pI	Number of Isoforms	pI after neuraminidase	pI after endo H
MN-rgp120	CHO	8.7	4.2 – 5.5	16	5.6 – 6.7	3.0 – 3.9
MN-rgp120	293	8.7	4.4 – 6.8	24	6.8 – 7.1	3.0 – 3.9
A244-rgp120	CHO	8.4	3.6 – 6.6	24	7.5 – 8.5	3.0 – 3.9
A244-rgp120	293	8.4	3.6 – 7.5	40	7.0 – 7.5	3.0 – 3.9

pI, indicates isoelectric point.

### Characterization of A244-rgp120 produced in CHO and 293 cells

**Figure 4:**
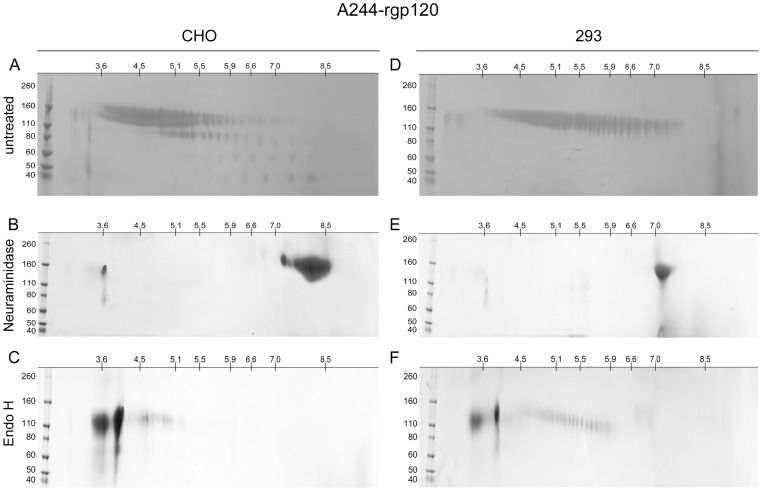
Isoelectric focusing of A244-rgp120 expressed in CHO and 293 cells with and without glycosidase digestion. The recombinant HIV-1 envelope proteins were left untreated (panels A and D), or digested with neuraminidase (panels B and E) or Endo H (panels C and F). For fractionation in the first dimension, the proteins were resolved by isoelectric focusing using 11 cm IPG strips (pI 3 to 10) (Bio-Rad Laboratories, Hercules, CA). For fractionation in the second dimension, the proteins were resolved using 4–15% tris glycine SDS-PAGE gels. Bands were visualized by Coomassie blue staining. Pre-stained molecular weight markers are shown to the left.

2D-PAGE analysis of the clade E A244 envelope proteins produced in CHO and 293 cells gave similar results (Figure 4A and D). The pIs of A244-rgp120 variants produced in both 293 and CHO cells were much more acidic than the predicted pI of 8.4 for A244-rgp120, suggesting that both proteins incorporated a significant amount of sialic acid. As with MN-rgp120, the protein produced in CHO cells was distinctively more acidic than the A244-rgp120 variants produced in 293 cells. However, a significantly greater number of pI variants was seen with both A244-rgp120 proteins than was seen with the MN-rgp120 variants (Table 1). Thus, 24 distinct isoforms could be resolved in the CHO protein with pIs ranging from 3.6 to 6.6, with the majority of protein possessing pIs between 3.6 and 5.9 (Figure 4A). In contrast, the protein produced in 293 cells had an even greater level of variation such that 40 distinct isoforms could be visualized (Figure 4D). These isoforms ranged in pI from 3.6 to 7.5. We observed some heterogeneity in molecular weight as well as charge, with the more acidic species, as expected, possessing slightly higher molecular weights due to the additional carbohydrate moieties required for sialic acid-containing N-linked carbohydrate. In addition, it was apparent that some proteolytic degradation had occurred in the archival samples of A244-rgp120, resulting in a plume of variants in the 70 kDa range. As with MN-rgp120, the pIs of the 293 cell proteins were more basic and contained more species than the proteins produced in CHO cells.

### Neuraminidase digestion of MN-gp120 and A244-gp120

In order to test the possibility that the variation of isoforms observed on 2D-PAGE gels was due to variation in the number of sialic acid residues, the four envelope proteins described above were treated with neuraminidase. The results of this study are shown in panels B and E of Figures 2 and 4. We found that neuraminidase treatment dramatically reduced the number of envelope glycoforms and caused a large shift in pI towards the basic range of the pH gradient. This result clearly indicated that much of the isoform heterogeneity observed in MN-rgp120 and A244-rgp120 was due to differences in the incorporation of sialic acid. Thus, before neuraminidase treatment, the MN-rgp120 produced in CHO cells possessed approximately 16 different species that ranged in pI between 4.2 and 5.1. After neuraminidase treatment, the material exhibited 8 isoforms ranging in pI from 5.6 to 6.7 (Table 1). Similarly, prior to neuraminidase digestion, MN-rgp120 produced in 293 cells exhibited 24 discrete isoforms with pIs in the 4.4 to 6.8 range. However, after neuraminidase digestion, approximately 5 isoforms were detected with pIs spanning the 6.8 to 7.3 range. These results demonstrate that much of the variation in pI between isoforms of MN-rgp120 produced in CHO cells and 293 cells is due to variation in the amount of sialic acid incorporated. Similar results were also observed in neuraminidase-treated A244-rpg120, with pI ranges of 7.5–8.5 and 7.0–7.5 for materials produced in CHO cells and 293 cells, respectively. In this study, we noted that after the neuraminidase digestion, pIs of both A244-rgp120 and MN-rgp120 produced in CHO cells and 293 cells were still lower than predicted pIs for non-glycosylated gp120 proteins, which indicated incomplete digestion. Incomplete digestion was not surprising, since the proteins were not reduced or denatured prior to neuraminidase digestion.

### Endo H digestion of MN-gp120 and A244-gp120

Endo H is an enzyme that is able to degrade the high mannose form of N-linked carbohydrate but has no effect on complex, sialic acid-containing N-linked carbohydrate. Endo H treatment of MN-rgp120 produced in CHO and 293 cells also resulted in a dramatic reduction of gp120 isoforms, but in this case the pIs were shifted to the opposite (acidic) end of the pH gradient (Panels C and F of Figures 2 and 4). Thus, treatment with Endo H eliminated most of the species found in the pI 4–5.9 range, and resulted in a large accumulation of isoforms in the pI 3.0–3.9 range. This result clearly indicated that all four envelope preparations possessed a high percentage of simple N-linked carbohydrate, and that Endo H-resistant complex carbohydrate was also present. Although multiple isoforms with pIs approaching 3.0 were observed, it should be noted that the IPG strips used for IEF have a lower pH limit of 3.0, and thus the resolution at the lower end of the pH gradient is limited. We also noted that Endo H-treated A244-gp120 produced in 293 cells consistently showed a greater number of Endo H-resistant bands than the A244-rgp120 produced in CHO cells, indicating greater complexity of complex carbohydrate structures. In the A244 Endo H digests (Figure 4, panels C and F), and to a lesser extent in the MN digests (Figure 2, panels C and F), we noted bands in the vicinity of pH 3.9 with molecular weights below 80 kDa. These bands appear to be gel artifacts, perhaps due to protein precipitation, which are not observed in the 1D SDS-PAGE gels. Together these results (Table 1) demonstrate that rgp120 proteins produced from CHO cells are more acidic and exhibit fewer isoforms than rgp120 proteins produced from 293 cells. Deletion of high mannose glycoforms with Endo H or sialic acid-containing glycoforms with neuraminidase shifts the rgp120 isoelectric point to more acidic or basic pH ranges, respectively.

### Effects of glycosylation on gp120 antigenicity

**Figure 5:**
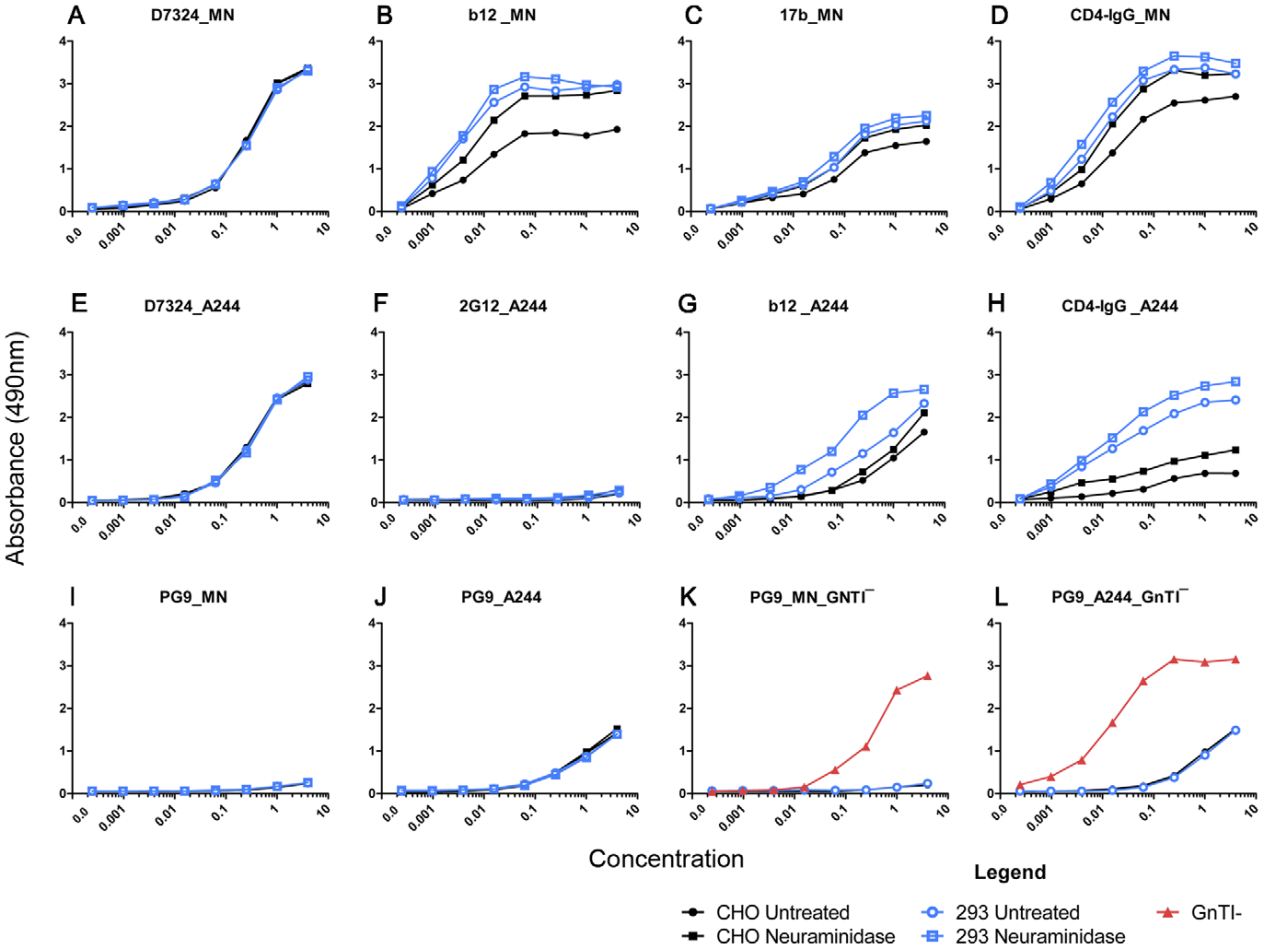
The binding of MAbs and CD4-IgG to differentially glycosylated forms of MN-rgp120 and A244-rgp120. Recombinant gp120s were expressed in CHO cells (black lines), 293 cells (blue lines), or GnTĪ 293 cells (red lines). Antibody binding to MN-rgp120 or A244-rgp120 was measured to untreated (circles) and neuraminidase-treated (squares) envelope proteins. Envelope proteins produced in GnTĪ 293 cells are indicated by triangles. The specific antibodies used in each experiment are identified in each panel. Proteins were coated directly onto microtiter plates, and binding was determined by ELISA.

The effect of glycoform heterogeneity on protein antigenicity was evaluated using a panel of neutralizing MAbs (b12, 17b, 48d, 2G12, PG9, and 447-52D) and the antiviral entry inhibitor, CD4-IgG, to test the MN- and A244-rgp120 proteins produced in CHO and 293 cells before and after treatment with neuraminidase. Epitopes affecting the CD4 binding site were evaluated using CD4-IgG and the b12, 17b, and 48d MAbs. Carbohydrate-dependent epitopes in the V2 and V3 domains were evaluated with the PG9 and 2G12 MAbs, respectively. Non-carbohydrate-dependent epitopes in the V3 domain were evaluated with the 447-52D MAb [32], [24]. A polyclonal serum (D7324) directed against a conserved sequence in the C5 domain was used as a positive control. No difference in antibody binding to MN-rgp120 produced in CHO or 293 cells was seen using the D7324 polyclonal serum (Figure 5A). Similarly, no difference in antibody binding was detected using the V3 domain-specific 447-52D MAb or the conformation-dependent 48d MAb (data not shown). No binding to MN-rgp120 produced in either cell line was detected with either the PG9 (5I) or 2G12 (data not shown) MAbs. However, several differences were noted in the binding of the bN CD4-blocking MAb, b12 (Figure 5B), the 17b antibody recognizing the CD4-induced (CD4i) conformation (Figure 5C), and CD4-IgG (Figure 5D). In each case, the binding of these probes was greater to MN-rgp120 produced in 293 cells, compared to CHO cells. To see if these differences could be due to differences in glycosylation, we treated the proteins with neuraminidase to remove terminal sialic acid. We found that treatment of MN-rgp120 produced in CHO cells increased the binding of b12, 17b and CD4-IgG. However, treatment of MN-rgp120 produced in the 293 cells had no effect. This result suggested that the difference in binding of ligands targeting the CD4 binding site was due to a difference in sialic content, and that the proteins expressed in CHO cells incorporated more sialic acid at positions affecting the CD4 binding site than proteins produced in 293 cells.

We next evaluated the binding of the MAbs and CD4-IgG to A244-rgp120 produced in CHO and 293 cells. As noted above, there was no difference in binding of the D7324 serum to A244-rgp120 expressed in either CHO or 293 cells (Figure 5E). We found that several of the MAbs in the panel, including 2G12 (Figure 5F), 447-52D, 48d, and 17b (data not shown), were unable to bind to the A244-rgp120 protein. This result was consistent with the presence of sequence polymorphisms at or near residues known to be important for the binding of these antibodies. When the binding of the b12 MAb and CD4-IgG was examined, significant differences were observed (Figures 5G and H). Both ligands bound significantly better to A244-rgp120 produced in 293 cells as compared to CHO cells. However, treatment with neuraminidase had little or no effect on the binding of these ligands to the CHO proteins, but increased binding to the proteins produced in 293 cells. This result suggested that CHO cells failed to incorporate sialic acid at glycosylation sites in A244-rgp120 that affected CD4 binding, whereas 293 cells did incorporate sialic acid at these sites. As noted for MN-rgp120, removal of sialic acid with neuraminidase increased the binding of b12 and CD4-IgG to A244-rgp120 produced in 293 cells.

When PG9 binding to MN-rgp120 was examined, we were unable to detect binding to the proteins expressed in either CHO or 293 cells (Figure 5I). This result was consistent with previous reports [20] that the MN strain of HIV-1 was resistant to neutralization by the PG9 MAb even though the MN-rgp120 envelope protein possessed potential N-linked glycosylation sites (PNGS) at positions 156 and 160 known to be essential for the binding of this MAb [24], [20]. In contrast, we observed modest, but significant binding to A244-rgp120 (Figure 5J). This result was consistent with recent studies [33] noting that A244-rgp120 was unusual in its ability to be recognized by several neutralizing antibodies to the V2 domain, including PG9 and CHO1-4. Since MN-rgp120, like A244-rgp120, possesses PNGS at positions 156 and 160, these results suggested that the type of carbohydrate present at these positions might explain the difference in PG9 binding that we observed. To test this possibility, we expressed the MN-rgp120 in 293 cells that are GnTI-deficient, resulting in mannose-5 structures at all PNGS. Previous studies have reported that PG9 preferentially recognized mannose-5 structures [20]. When MN-rgp120 produced in GnTĪ cells was evaluated, we found that PG9 binding was greatly improved with a 50% effective concentration (EC50) of approximately 0.1 μg/mL (Figure 5K). When A244-rgp120 was grown in the GnTĪ cells, binding also improved dramatically with an EC50 of approximately 0.01 μg/mL (Figure 5L). These results demonstrated that the inability of PG9 to bind to MN-rgp120, and its modest binding to A244-rgp120, was due to suboptimal glycosylation in CHO and 293 cells. We found that PG9 binding to both MN- and A244-rgp120 could be greatly improved by production in a cell line where the carbohydrate structures were limited to mannose-5 glycans. However, since there was a 10-fold difference in the EC50 between MN-rgp120 and A244-rgp120 produced in GNTĪ cells, something other than the glycan structure, perhaps the V2 domain conformation, also affects the PG9 binding to these antigens.

## Discussion

The studies described in this paper show that the MN-rgp120 and A244-rgp120 immunogens, contained in the AIDSVAX B/E vaccine [3], [4] and used in the VAX003 and RV144 Phase 3 trials [6], [1], consisted of a complex swarm of isoforms that differed in isoelectric point, net charge, and carbohydrate structure. Although it has long been known that the carbohydrate composition of recombinant gp120 is heterogeneous [34], [17], [9], [35], the number of discrete species with different isoelectric points has not previously been described and was larger than expected. It is not clear to what extent the glycosylation in recombinant protein immunogens resembles the glycosylation of gp120 *in vivo*. It is likely, however, that glycosylation varies as a function of cell type, expression level, and whether the envelope protein is incorporated on the surface of cells or the surface of virions. Our data demonstrated that the glycan shield is determined not only by whether a PNGS is utilized, but also by the specific structure of the carbohydrate attached to each site. In theory, viruses *in vivo* could escape neutralization by antibodies such as PG9 and 2G12 by at least three distinct mechanisms: 1) mutations that destroy the NXS/T motif required for N-linked glycosylation, as in the case of the 2G12 sites in MN- and A244-rgp120s; 2) sequences that favor the incorporation of more complex carbohydrate structures, as in the case of MN-rgp120 compared to A244-rgp120; or 3) a selection process whereby cells with enhanced ability to synthesize sialic acid-containing glycoforms are able to evade neutralization by antibodies such as PG9 that require specific high mannose structures for binding.

The recombinant gp120 proteins in the AIDSVAX B/E vaccine used in the RV144 trial were expressed with a flag epitope derived from herpes simplex virus glycoprotein D (HSV-gD) [36], [4]. The flag epitope provided the means by which recombinant envelope proteins from different strains (e.g. MN- and A244-rgp120) could be purified by a generic immunoaffinity purification process [3]. The immunoaffinity process does not involve selection of any particular glycan species. In contrast, the purification process used in the production of other candidate gp120 vaccines [37]–[39] often depends on the use of lectins that recognize specific high mannose carbohydrate structures. Thus the spectrum of glycan structures found in the AIDSVAX B/E vaccine used in the RV144 trial is unlikely to be replicated in vaccines purified by lectin affinity chromatography, even though both are produced in CHO cells. Besides the purification process, it is also known that fermentation conditions can affect the specific type of carbohydrate structures incorporated into recombinant proteins [40], [29], [41]. Thus, glucose starvation, depletion of key amino acids such as glutamine, accumulation of pyruvate leading to low pH, and accumulation of ammonium ion are all factors known to affect the extent of incorporation of terminal sialic acid residues. Although we do not know the extent to which glycoform heterogeneity in the gp120s contained in the AIDSVAX B/E vaccine affected the protective immunity observed in the RV144 trial, our results suggest that glycosylation heterogeneity may be a significant variable that deserves further investigation.

Our current studies demonstrated that glycan structure affects the antigenicity of some epitopes (e.g. the CD4 binding site and the PG9 epitope in the V2 domain) more than others. The relevance of gp120 glycosylation to the binding and development of bN antibodies has recently been the subject of considerable attention [42], [13], [15], [31], [16]. Kong et al. [15] studied the antigenicity and immunogenicity of wild-type rgp120, and gp120 produced under conditions that prevented the incorporation of sialic acid. They reported that few significant differences were observed in the binding of MAbs to wild type gp120 and gp120 treated with the glycosylation inhibitor, kifunensine. A significant difference was noted in the binding of CD4-IgG and the bN b12 MAb that recognizes an epitope that overlaps the CD4 binding site [15]. In the present studies, we likewise observed enhanced binding of CD4-IgG and the b12 MAb to gp120 variants with reduced levels of sialic acid resulting from expression in 293 cells or treatment with neuraminidase. The effect was envelope-specific and greater for A244-rgp120 than MN-rgp120. In addition, the magnitude of the CD4–IgG binding effect (3-fold and 9-fold difference for MN-rgp120 and A244-rgp120 respectively) was comparable to the 6-fold difference in CD4-IgG binding described in the earlier study (3). However, the effect we observed on the binding of the b12 MAb (at most a 9-fold difference) was far lower than the 82-fold difference in b12 binding reported for envelope proteins lacking sialic acid that were produced by treatment with kifunensine [15]. Binley et al. [42] reported that pseudoviruses with fully trimmed oligomannose cores produced in GnTĪ 293 cells exhibited increased neutralization sensitivity to soluble CD4, CD4-IgG, and antibodies to the V3 domain, but not to the 2G12 and b12 MAbs. Eggink et al. [14] expressed LAI, JF-RL, and KNH1144 monomeric gp120s, and JR-FL gp140 trimers in GnTI-deficient 293 cells, and reported no difference in binding using the tetravalent CD4-IgG2 and the b12 MAb. We are unable to explain the difference in results between these two studies.

It has long been known that some bN MAbs (e.g. 2G12) recognize epitopes involving carbohydrate structures [43]. However, the 2G12 glycosylation sites are not well conserved and both the MN-rgp120 or A244-rgp120 immunogens possess common polymorphisms at position 332 (Ile for MN-rgp120 and Glu for A244-rgp120) that preclude the binding of this antibody. Recently, several potent bN MAbs (PGT 127 and 128) have been described [44] that preferentially recognize mannose-8 and mannose-9 structures in positions N301 and N332 for antibody binding [45]. In the present studies, we observed that the potent bN MAb, PG9, exhibited moderate binding to A244-rgp120 expressed in either CHO or 293, but little if any binding to MN-rgp120. This result was surprising, since unlike the situation with the 2G12 MAb, both proteins possesses PNGS at positions 156 and 160 required for the binding of the PG9 MAb [20], [46]. Based on the recent paper showing that approximately 70% of the interface surface area for PG9 binding to V1/V2 domain was contributed by the mannose-5 glycans at positions N156 and N160 [20], we reasoned that the difference in PG9 binding to MN- and A244-rgp120 might be due to differences in the specific glycan structures attached at positions 156 and 160. This hypothesis was confirmed by the results obtained with MN-rgp120 produced from GnTI-deficient 293 cells, which limited the structure of glycans at PNGS to Man_5_GlcNAc_2_ (mannose-5) structures.

Thus the ability of PG9 to bind to envelope proteins is determined by both the presence of PNGS at position 156 and 160 [24] and the specific glycan structures attached to these sites as is the case for MN-rgp120 and A244-rgp120. In view of these results, it is important to understand the factors that determine the type of glycosylation attached to each site. Unfortunately these factors are not well understood. It is known that the extent of glycosylation varies between cell types from the same species [47], [31]. It is also known that there is an inverse relationship between protein expression levels and the incorporation of complex carbohydrate. Several lines of evidence suggest that this relationship is attributable to the saturation of trans-Golgi glycosyltransferases or the depletion of UDP- and CMP-monosaccharide precursor pools [48]–[50]. However, it is unlikely that a difference in expression levels accounts for the difference in PG9 binding to A244- and MN-rgp120 in 293 cells, since both proteins were expressed at similar levels.

Besides the differences in the binding of bNAbs such as PG9, the differences in net charge might affect the antigen processing and presentation required for T-cell recognition that occurs at low pH in endosomal and lysosomal compartments [51], [52]. The low pI (pH 4–5) isoforms of rgp120 are predicted to carry a net negative charge in humoral circulation (pH = 7.4), but then become uncharged when they encounter lysosomal pH (pH = 4.5). In contrast, the high pI (pH 6–8) isoforms are predicted to carry a neutral charge in humoral circulation, but become positively charged after being taken into the lysosome. These differences in charge might alter antigen processing and/or presentation that may alter intracellular binding to major histocompatibility complex II (MHCII) molecules and subsequent recognition by T-cells as well as B-cells.

Our studies do not allow us to know whether the heterogeneous glycosylation pattern found in MN-rgp120 and A244-rgp120 is an advantage or disadvantage for envelope-based HIV vaccines. On one hand, mannose-5 structures such as the type found on proteins grown in GNTĪ cells would be expected to favor the formation and binding of PG9-like antibodies. On the other hand, other potent carbohydrate-dependent neutralizing MAbs such as 2G12, PGT 127, and PGT 128 favor the larger Man_9_GlcNAc_2_ (mannose-9) structures [21] induced by the mannosidase I inhibitor, kifunensine, but bind poorly to the mannose-5 structures. In this regard, perhaps a vaccine cocktail including the same envelope protein produced with mannose-5 structures and mannose-9 structures would be an advantage in the productions of bNAbs. Although its unusual ability to bind PG9-like antibodies [33] suggests that the A244-rgp120 would be better than MN-rgp120 in eliciting PG9-like antibodies, no evidence of broadly neutralizing PG9-like activity was observed in sera from the RV144 or VAX003 clinical trials, even though robust immune responses to the V2 domain of A244-rgp120 were detected [6], [53]. Indeed, we do not know whether immunization schedules practical for human vaccines can stimulate the formation of antibodies to the epitopes recognized by rare bNAbs such as PG9, 2G12, and b12. It is possible that only chronic infection provides sufficient time and exposure required for the selection of the highly evolved and extensively rearranged immunoglobulin structures characteristic of the majority of bN-MAbs identified to date [54]. The studies described in this paper demonstrate that recombinant envelope proteins produced in mammalian cells represent a diverse mixture of high mannose and sialic acid-containing isoforms. In principle, we would expect the best vaccine might be a mixture of gp120 variants that replicated all of the potential glycan structures likely to be incorporated by the envelope proteins responsible for virus-mediated and cell-to-cell infection. The complexity of N-linked glycosylation in recombinant proteins represents a challenging analytical and manufacturing problem, as well as a biological problem. In order to verify and replicate the RV144 trial result in other populations, one would want to limit the number of variables. Therefore it would seem prudent to develop a production cell line and purification process that as closely as possible replicates the variation in glycan structure and net charge seen with the AIDSVAX B/E vaccine. While this may be difficult, the IEF system described in this paper provides a simple and practical way to characterize the complexity of glycoforms in cell lines created to produce HIV-1 envelope proteins for vaccine studies.
